# Efficacy and safety of Wenxin Keli for ventricular premature beat: An overview of systematic reviews and meta-analyses

**DOI:** 10.1097/MD.0000000000047265

**Published:** 2026-01-16

**Authors:** Xia Zhou, Wenhua Zhou, Biao Luo, Lvyu Deng, Daohui Gan, Xiaohan Wu, Fengya Zhu, Liuying Li

**Affiliations:** a Traditional Chinese Medicine Department, Zigong First People’s Hospital, Zigong, Sichuan, China.

**Keywords:** clinical symptoms, meta-analyses, overview, systematic reviews, ventricular premature beat, Wenxin Keli

## Abstract

**Background:**

Wenxin Keli (WXKL) is a common medication used to treat ventricular premature beat (VPB). As a complementary alternative therapy, the clinical efficacy and safety of WXKL-assisted treatment of VPB has been reported by several systematic reviews (SRs), but the quality of evidence has not been systematically reviewed.

**Methods:**

We conducted a literature search on 7 databases until May 1, 2025. Risk of bias in systematic was used to assess the risk of bias in SRs. A measurement tool to Assess Systematic Reviews 2 was used to evaluate the methodological quality of the SRs, respectively. Grading of recommendations assessment, development and evaluation (GRADE) was used to evaluate the quality grading of outcomes.

**Results:**

Eleven SRs, including 211 clinical randomized controlled trials and 19,673 patients, met the inclusion criteria. Four SRs (36.36%) were rated as low risk and 7 SRs (63.64%) were rated as high risk. Only 3 SRs had low methodological quality, while the remaining 8 SRs had critically low methodological quality. We evaluated 46 outcomes, with moderate quality evidence in 6 (13.04%), low-quality evidence in 24 (52.17%), very low-quality evidence in 16 (34.78%). The pooled results suggest that WXKL appears to have an advantage in improving 24-hour Holter electrocardiogram and clinical symptoms in VPB, but the overall efficacy in VPB should be interpreted with caution. Meta-analysis showed that WXKL combined with conventional western medications improved the total effective rate (Relative risk [RR] = 1.08, 95% confidence interval [CI]: 1.06–1.11, *P* < .00001), the effect of 24-hour Holter electrocardiogram (RR = 1.22, 95% CI: 1.17–1.27, *P* < .00001) and clinical symptoms (RR = 3.00, 95% CI: 2.17–4.27, *P* < .00001). There were no significant differences in adverse effects (RR = 0.85, 95% CI: 0.68–1.06, *P* = .16).

**Conclusion:**

WXKL adjuvant therapy of VPB may be effective, but the evidence quality is low. Physicians should choose carefully in the context of the clinical situation when referring to SRs of WXKL for the treatment of VPB for clinical decision making.

## 1. Introduction

Ventricular premature beat (VPB) is a premature contraction of the ventricle produced by the early depolarization of ectopic foci of excitation in the ventricular muscle below the bundle of Hirschsprung and branches. VPB has a detection rate of 1 to 3%^[1]^ in the general population and is one of the most common clinical arrhythmias. Some benign ventricular prematurities also have the risk of inducing malignant arrhythmias such as polymorphic ventricular tachycardia and ventricular fibrillation, while frequent ventricular prematurities can lead to the impairment of ventricular systolic function and even arrhythmogenic cardiomyopathy.^[2]^

Currently, the treatment of VPB is based on anti-arrhythmic drugs such as beta blockers, mexilol, propafenone, verapamil and catheter ablation.^[3]^ However, antiarrhythmic drugs may cause new arrhythmias such as bradycardia and atrioventricular block and have toxic side effects. Catheter ablation is indicated for patients who have failed drug therapy or combined with premature ventricular cardiomyopathy,^[4,5]^ but it is an invasive treatment with surgical risks,^[6]^ resulting in poor patient compliance.

Therefore, there is a need to find potential ways to relieve VPB. Wenxin Keli (WXKL) is a Chinese medicinal preparation for VPB by Shandong Buchang Pharmaceutical Co. It is composed of *Polygonati rhizoma* (Huang Jing), *Codonopsis radix* (Dang Shen), *Ambrum* (Hu Po), *Notoginseng Radix et Rhizoma* (San Qi) and *Nardostachyos Radix et Rhizoma* (Gan Song). Clinical studies have shown that WXKL may suppress sarcoplasmic reticulum Ca^2+^ release and maintain intracellular Ca^2+^ balance,^[7]^ regulate mitochondrial function and homeostasis,^[8]^ regulate the CaMK II signal transduction pathway to inhibit arrhythmia and improve cardiac function.^[9]^ Accumulating evidence from various animal and cell studies has shown that WXKL could protect the myocardium and anti-arrhythmia against cardiovascular diseases. WXKL exhibited its cardioprotective roles by inhibiting the inflammatory reaction, decreasing oxidative stress, regulating vasomotor disorders, lowering cell apoptosis and protecting against endothelial injury, myocardial ischemia, cardiac fibrosis and cardiac hypertrophy.^[10]^

Although WXKL has a demonstrated pharmacological basis and shows promising clinical efficacy in treating VPB, its evidence level remains to be fully established. Overview is the synthesis of current evidence from multiple relevant systematic reviews (SRs) to obtain more focused, high-quality evidence.^[11]^ This study conducted an overview of SRs and meta-analyses on the efficacy and safety of WXKL for VPB, with the objective of evaluating the strength and reliability of the available evidence. This overview provides a critical synthesis of existing evidence, consolidating and clarifying the therapeutic profile of WXKL. By affirming its efficacy and confirming its favorable safety record for VPBs, it offers clinicians a consolidated, high-level evidence base to support informed treatment decisions.

## 2. Methods

Systematic review registration: PROSPERO, http://www.crd.york.ac.uk/PROSPERO/, identifier (CRD42023448011).

### 2.1. Inclusion criteria

#### 2.1.1. Types of reviews

SRs and meta-analyses of clinical randomized controlled trials (RCTs) were included. The language was limited to Chinese and English.

#### 2.1.2. Types of participants

The participants met the recognized diagnostic criteria for VPB, regardless of gender, age, race, time of onset and source of cases.

#### 2.1.3. Types of Interventions

The intervention strategy in the treatment group was WXKL combined with conventional therapy (CT). For the control group, CT was used as an intervention method.

#### 2.1.4. Types of outcomes

Studies using at least one of the following outcome indicators were included Primary efficacy outcome indicators: Effect of 24-hour Holter electrocardiogram; Clinical efficiency rate; Secondary efficacy outcome indicators: Effect of palpitation symptom; Effect of chest distress symptoms; Drug safety evaluation; and Safety outcome indicators: Adverse events including bradycardia, atrioventricular block, gastrointestinal intolerance, itchy skin, or rash.

### 2.2. Exclusion criteria

WXKL was not the only adjuvant treatment in the experimental group; Duplicate publications or restricted access to literature; The data are incomplete and the full text of the literature cannot be obtained; Other types of research, such as animal experiments, protocols, conference papers and case reports; and Systematic review without meta-analysis and network meta-analysis.

### 2.3. Search strategy

We searched 3 international databases (PubMed, Cochrane Library and Embase) and 4 Chinese databases (CNKI, SinoMed, Wanfang and VIP) to identify eligible SRs published up to May 1, 2025, without language restriction. We used a combination of subject words and free words, including “VPB,” “premature ventricular contraction,” “ventricular premature contraction,” “VPB,” “Wen-xin-Ke-Li,” “WXKL,” “Wenxin,” “SR,” “systematic evaluation” and “meta-analysis.” In addition, we manually searched the list of references in the included SRs. Search strategies are detailed in the Supplementary Materials (Supplemental Digital Content, https://links.lww.com/MD/R172).

### 2.4. Study selection and data extraction

According to the comprehensive retrieval strategy, 2 reviewers independently conducted literature retrieval and screening. The opinion of a third reviewer was sought when there was disagreement. After identifying eligible studies, 2 researchers independently extracted relevant data according to standardized extraction tables, such as author, publication year, sample size, diagnostic criteria, interventions, outcomes, adverse reactions, conclusions etc. Two reviewers cross-checked the extracted content and a third reviewer was consulted to resolve any discrepancies.

### 2.5. Assessment of SRs

SRs that met the inclusion criteria were independently assessed by 2 reviewers for the methodological quality of the SRs, the quality of evidence and the risk of bias.

#### 2.5.1. ROBIS

We used the risk of bias in systematic (ROBIS) tool^[12]^ to assess the risk of bias for SRs, including 4 key areas: study eligibility criteria, study identification and selection, data collection and study appraisal, and synthesis and findings. Finally, we divided the risk level into “low risk,” “high risk” and ‘unclear risk’. One person assessed ROBIS, another person checked this assessment and then both reviewers discussed the results. In the case of a disagreement, a third party was consulted.

#### 2.5.2. AMSTAR 2

Two reviewers used A measurement tool to Assess Systematic Reviews 2 (AMSTAR 2)^[13]^ to evaluate the methodological quality of SRs. This tool includes 16 items in total, with items 2, 4, 7, 9, 11, 13, and 15 considered key items. Items 2, 4, 7, 8, and 9 are rated as yes, no or partially yes. The study quality was judged as high when no or only one non-essential item did not meet the requirements, medium when more than one non-essential item did not meet the requirements, low when any of the essential items did not meet the requirements and very low when more than one essential item did not meet the requirements.

#### 2.5.3. GRADE

Two researchers independently used the grading of recommendations assessment, development and evaluation (GRADE) tool^[14]^ to evaluate the quality of the evidence. The tool includes 5 aspects: risk of bias, inconsistency, indirectness, imprecision and publication bias. We graded the quality of evidence as “high,” “moderate,” “low,” or “very low.” The 2 reviewers cross-checked the results and disputes were resolved by a third reviewer.

### 2.6. Data analysis

RevMan5.3 is used for statistical analysis of the data. Relative risk (RR) was used as the statistic for categorical variables, for continuous variables, mean difference (MD) was used as the statistic if the measurement method and units were the same and standardized mean difference (SMD) was used as the statistic if measured by different methods or with different units and its 95% confidence interval (CI) was calculated. Heterogeneity was tested using the *I*^2^ quantitative method. If *I*^2^ ≤ 50%, homogeneity was considered good and Meta-analysis was performed using a fixed-effects model; if *I*^2^ > 50%, statistical heterogeneity between studies was indicated and a meta-analysis was performed using a random-effects model and, if necessary, subgroup analysis or sensitivity analysis. Publication bias was judged using funnel plots.

## 3. Results

### 3.1. Search results

**Figure 1.**
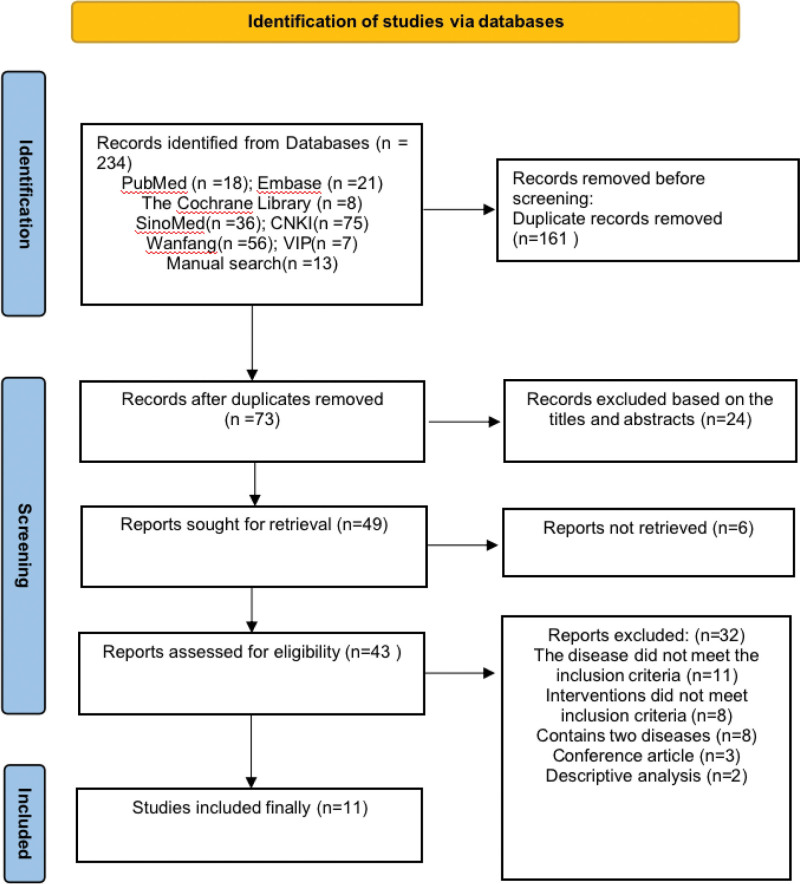
The detailed flow chart.

We retrieved 234 related studies from the 7 databases. Of these, we deleted 161 duplicates and then screened 73 studies, followed by full-text evaluation. Finally, we included 11 SRs. The detailed flow chart is shown in Figure 1. The list of exclusions and reasons are shown in the Supplementary Materials (Supplemental Digital Content, https://links.lww.com/MD/R172).

### 3.2. Characteristics of included SRs

Eleven SRs,^[15–25]^ published from 2013 to 2025, including 211 RCTs and 19,673 patients, met the inclusion criteria. 8 SRs specified diagnostic criteria, 3 SRs^[23–25]^ used routine electrocardiogram, 2 SRs^[15,18]^ used expert consensus on ventricular arrhythmia, 3 SRs^[19–21]^ used routine electrocardiogram or 24-hour Holter electrocardiogram and the remaining SRs did not mention specific diagnostic criteria. The control group usually received CT of amiodarone, metoprolol or propafenone, while the treatment group was combined with WXKL on the basis of control group. Regarding the methodological quality assessment of SR, 4^[15,16,22,23]^ used the Jadad scale and the remaining 7 used the Cochrane Collaboration Network risk of bias assessment tool. The basic characteristics of the literature are shown in Table 1.

**Table 1:** The basic characteristics of the literature.

Included studies	Number of RCTs (participants)	Diagnostic criteria	Intervention	Comparison	Adverse reaction	Primary outcomes	Methodological evaluation tool	Main conclusion
Shen J (2016)^[15]^	10 (488/450)	③	WXKL + metoprolol	Metoprolol	Unclear	①②③④	Jadad	WXKL combined with betaloc can improve the clinical symptoms and Holter efficacy of VPB patients, with similar adverse reactions.
Du H (2014)^[16]^	20 (1029/956)	Unclear	WXKL + CT	CT	Unclear	①②④	Jadad	Compared with CT, WXKL can improve clinical efficacy, improve ECG and reduce adverse reactions.
Sun LH (2013)^[17]^	69 (2788/2661)	Unclear	WXKL + CT	CT	①②③	①②④	Cochrane	WXKL is safe and effective in the treatment of VPB.
Huang P (2022)^[18]^	11 (477/477)	③	WXKL+ metoprolol	Metoprolol	③④⑤	①②④	Cochran	WXKL increased the efficacy of the drug, reduced the occurrence of adverse reactions.
He M (2016)^[19]^	10 (1134/1034)	① or ②	WXKL + CT	CT	①③⑤	②③④	Cochran	WXKL is a alternative and complementary medicine for VPB.
Liu XS (2018)^[20]^	17 (755/732)	① or ②	WXKL + amiodarone	Amiodarone	②④⑤	①④⑤⑥	Cochrane	WXKL combined with amiodarone in the treatment of VPB is better than amiodarone alone, with less adverse reactions.
Li JK (2016)^[21]^	21 (1234/1108)	① or ②	WXKL	Propafenone	①②④	②③④	Cochrane	WXKL can improve the efficacy of comprehensive symptoms, reduce the incidence of adverse reactions.
Guo RT (2016)^[22]^	20 (787/781)	Unclear	WXKL + metoprolol	Metoprolol	②③④	②③④	Jadad	WXKL combined with metoprolol can improve clinical symptoms and reduce VPB.
Liu Q (2015)^[23]^	12 (518/503)	①	WXKL + CT	CT	④⑤	①②④	Jadad	WXKL combined with metoprolol for the treatment of VPB was better than the control group, and its safety was good.
Li M (2017)^[24]^	8 (319/316)	①	WXKL + CT	CT	①②④	①⑧	Corhrane	WXKL may be effective and safe for treating VPB and HF.
Zheng R (2018)^[25]^	13 (570/556)	①	WXKL + amiodarone	Amiodarone	④⑥	①④⑦	Corhrane	WXKL + amiodarone is safe more effective compared with amiodarone alone.

Diagnostic criteria: ① Electrocardiogram; ② 24 h Holter electrocardiogram; ③ Expert consensus on ventricular arrhythmias.

Adverse reaction: ① Dizziness; ② Gastrointestinal reactions; ③ Hypotension; ④ Sinus bradycardia; ⑤ Atrioventricular block; ⑥ Sinus arrest.

Primary outcomes: ① Total effective rate; ② Effect of 24 h Holter electrocardiogram; ③ Clinical symptom improvement; ④ Adverse reaction; ⑤ Incidence of gastrointestinal reactions; ⑥ Incidence of atrioventricular block; ⑦ Heart rate; ⑧ Number of VPB reductions.

CT = conventional therapy, ECG = electrocardiogram, HF = heart failure, RCT = randomized controlled trial, VPB = ventricular premature beat, WXKL = Wenxin Keli.

### 3.3. Risk of bias

The ROBIS results for 11 SRs showed that 4 (36.36%)^[18,19,24,25]^ were rated as low risk and 7 (63.64%)^[15–17,20–23]^ were rated as high risk. The detailed results are shown in Table 2.

**Table 2:** The detailed results of ROBIS.

Review	Phase 2				Phase 3
	Study eligibility criteria	Identifification and selection of studies	Data collection and study appraisal	Synthesis and fifindings	Risk of bias in the review
Shen J (2016)^[15]^	Low risk	High risk	High risk	Low risk	High risk
Du H (2014)^[16]^	Low risk	High risk	High risk	Low risk	High risk
Sun LH (2013)^[17]^	Low risk	High risk	High risk	High risk	High risk
Huang P (2022)^[18]^	Low risk	Low risk	Low risk	Low risk	Low risk
He M (2016)^[19]^	Low risk	Low risk	high risk	Low risk	Low risk
Liu XS (2018)^[20]^	Low risk	Low risk	Low risk	Low risk	Low risk
Li JK (2016)^[21]^	Low risk	Low risk	High risk	High risk	High risk
Guo RT (2016)^[22]^	Low risk	High risk	Low risk	High risk	High risk
Liu Q (2015)^[23]^	Low risk	Low risk	High risk	High risk	High risk
Li M (2017)^[24]^	Low risk	Low risk	High risk	Low risk	Low risk
Zheng R (2018)^[25]^	Low risk	Low risk	High risk	Low risk	High risk

ROBIS = risk of bias in systematic.

### 3.4. Methodological quality

We used AMSTAR 2 to assess the methodological quality of the 11 SRs. Only 3 SRs had low methodological quality, while the remaining 8 SRs had critically low methodological quality.

Among the critical items, 2 SRs^[18,25]^ reported study protocols (item 2), one SR^[24]^ provided an exclusion list (item 7), all conducted a comprehensive database search but did not search in detail for possible potential literature (item 4), all used reasonable tools to assess the risk of bias for inclusion in the study literature (item 9), 10 SRs conducted data synthesis (item 11), 9 SRs considered the risk of bias (item 13) and 8 SRs fully investigated and discussed the possible impact of publication bias on the study results (item 15). Among the non-critical items, the SRs were better in terms of inclusion criteria (item 1), literature screening and data extraction (items 5 and 6), basic characteristics (item 8) and the discussion of heterogeneity (item 14). However, none of the SRs gave an explanation of the basis for the selection of the study design (item 3) and only one SR^[24]^ reported the source of funding (item 10); Seven SRs considered the potential impact of risk of bias on the quality of evidence (item 12) and 4 SRs^[18,19,24,25]^ declared that there was no conflict of interest (item 16). The detailed results are shown in Table 3.

**Table 3:** The detailed results of AMSTAR-2.

Included studies	AMSTAR-2																Quality
	Item 1	Item 2	Item 3	Item 4	Item 5	Item 6	Item 7	Item 8	Item 9	Item 10	Item 11	Item 12	Item 13	Item 14	Item 15	Item 16	
ShenJ (2016)^[15]^	Y	N	N	PY	Y	Y	N	PY	PY	N	Y	Y	Y	Y	Y	N	Critically low
Du H (2014)^[16]^	Y	N	N	PY	Y	Y	N	N	PY	N	Y	N	N	N	Y	N	Critically low
Sun LH (2013)^[17]^	Y	N	N	PY	N	N	N	N	PY	N	Y	Y	Y	Y	Y	N	Critically low
Huang P (2022)^[18]^	Y	Y	N	PY	Y	Y	N	Y	Y	N	Y	Y	Y	Y	Y	Y	low
He M (2016)^[19]^	Y	N	N	PY	Y	Y	N	Y	Y	N	Y	Y	Y	Y	Y	Y	Critically low
Liu XS (2018)^[20]^	Y	N	N	PY	Y	Y	N	PY	Y	N	Y	N	Y	Y	N	N	Critically low
Li JK (2016)^[21]^	Y	N	N	PY	Y	Y	N	PY	Y	N	Y	Y	N	N	Y	N	Critically low
Guo RT (2016)^[22]^	Y	N	N	PY	Y	Y	N	PY	Y	N	Y	N	Y	Y	N	N	Critically low
Liu Q (2015)^[23]^	Y	N	N	PY	Y	Y	N	PY	Y	N	N	N	Y	Y	N	N	Critically low
Li M (2017)^[24]^	Y	N	N	PY	Y	Y	PY	PY	Y	Y	Y	Y	Y	Y	Y	Y	Low
Zheng R (2018)^[25]^	Y	Y	N	PY	Y	Y	N	PY	PY	N	Y	Y	Y	Y	Y	Y	Low

AMSTAR-2 = a measurement tool to Assess Systematic Reviews 2, N = no, PY = partial yes, Y = yes.

### 3.5. Quality of evidence

Eleven SRs were included in this paper, but only one SR^[24]^ distinguished between primary and secondary indicators. The GRADE system was used to grade the quality of evidence for 46 outcome indicators. The grading results showed moderate quality evidence in 6 (13.04%), low-quality evidence in 24 (52.17%), very low-quality evidence in 16 (34.78%) and no high-quality evidence yet. The main influencing factors of the downgrade are as follows: Risk of bias, Publication bias, Inconsistency and Inaccuracy. The detailed results are shown in Table 4.

**Table 4:** The detailed results of GRADE.

Included studies	Outcomes	Number of RCTs (participants)	Certainty assessment					Effect estimate (95% CI)	*P*-value	Quality of evidence
			Risk of bias	Inconsistency	Indirectness	Imprecision	Publication bias			
Shen J (2016)^[15]^	Total effective rate	4 (268/235)	−1	0	0	0	−1	RR = 1..24 (1.15, 1.34)	<.001	Low
	Clinical symptom improvement	7 (332/326)	−1	0	0	0	−1	RR = 1..23 (1.15, 1.32)	<.001	Low
	Effect of 24 h Holter ECG	6 (220/215)	−1	0	0	0	−1	RR = 1..31 (1.19, 1.44)	<.00001	Low
	Adverse reaction	8 (366/330)	−1	0	0	0	0	RR = 0..77 (0.42, 1.18)	.18	Moderate
Du H (2014)^[16]^	Total effective rate	20 (1029/956)	−1	0	0	0	−1	0R = 3.72 (2.88, 4.81)	<.00001	Low
	Effect of 24 h Holter ECG	8 (384/330)	−1	0	0	0	−1	0R = 2.7 (1.88, 3.89)	<.00001	Low
	Adverse reaction	11 (570/547)	−1	0	0	0	−1	0R = 0.27 (0.19, 0.39)	<.00001	Low
Sun LH (2013)^[17]^	Total effective rate	63 (unclear)	−1	−1	0	0	−1	RR = 1.18 (1.14, 1.23)	Unclear	Very low
	Effect of 24 h Holter ECG	6 (unclear)	−1	0	0	0	−1	RR = 1.16 (1.05, 1.27)	Unclear	Low
	Adverse reaction	37 (unclear)	−1	0	0	0	−1	RR = 0..51 (0.37, 0.7)		Low
	Curative effect of TCM syndrome	4 (unclear)	−1	−1	0	−1	−1	RR = 1.52 (1.13, 2.04)	Unclear	Very low
	Palpitation symptom	8 (unclear)	−1	0	0	−1	−1	RR = 1.29 (1.19, 1.39)	Unclear	Very low
	Chest tightness symptom	6 (unclear)	−1	0	0	−1	−1	RR = 1.49 (1.27, 1.75)	Unclear	Very low
	Angina pectoris effect	5 (unclear)	−1	0	0	−1	−1	RR = 1.19 (1.09, 1.29)	Unclear	Very low
	ECG	5 (unclear)	−1	0	0	−1	−1	RR = 1.11 (0.95, 1.3)	Unclear	Very low
Huang P (2022)^[18]^	Total effective rate	11 (429/326)	−1	0	0	0	−1	RR = 1.32 (1.24, 1.40)	<.00001	Low
	Effect of 24 h Holter ECG	11 (429/326)	−1	0	0	0	0	RR = 1.32 (1.23, 1.41)	<.00001	Medium
	Adverse reaction	11 (429/326)	−1	0	0	0	−1	RR = 0.61 (0.35, 1.05)	.08	Medium
He M (2016)^[19]^	Effect of 24 h Holter ECG	10 (1034/1134)	−1	−1	0	0	−1	RR = 1.16 (1.00, 1.35)	.05	Very low
	Clinical symptom improvement	8 (954/1044)	−1	−1	0	0	−1	RR = 1.28 (0.96, 1.70)	.10	Very low
	Adverse reaction	8 (954/1044)	−1	0	0	0	−1	RR = 0.59 (0.35, 1.01)	.05	Low
Liu XS (2018)^[20]^	Total effective rate	17 (755/736)	−1	0	0	0	−1	OR = 3.52 (2.63, 4.71)	<.00001	Low
	Number of VPB reductions	5 (222/222)	−1	−1	0	0	−1	WMD = −315.59 (−441.76, −189.43)	<.00001	Very low
	Adverse reaction	8 (320/328)	−1	0	0	0	−1	OR = 0.26 (0.15, 0.45)	<.00001	Low
	Incidence of gastrointestinal reactions	8 (320/328)	−1	0	0	0	−1	OR = 0.33 (0.16, 0.69)	.003	Low
	Incidence of atrioventricular block	4 (170/176)	−1	0	0	−1	−1	OR = 0.22 (0.07, 0.71)	.01	Very low
Li JK (2016)^[21]^	Clinical symptom improvement	4 (244/198)	−1	0	0	−1	−1	OR = 2.65 (1.65, 4.24)	.0001	Very low
	Effect of 24 h Holter ECG	7 (466/401)	−1	0	0	0	−1	OR = 1.42 (1.01, 1.98)	.04	Low
	Palpitation effect	3 (206/175)	−1	0	0	−1	−1	OR = 4..44 (2.56, 7.22)	.00001	Very low
	Efficacy of chest tightness	3 (205/188)	−1	0	0	−1	−1	OR = 3.39 (1.96, 5.84)	.0001	Very low
	Adverse reaction	7 (466/401)	−1	0	0	0	−1	OR = 0.34 (0.20, 0.56)	.0001	Low
Guo RT (2016)^[22]^	Clinical symptom improvement	10 (356/347)	−1	0	0	0	0	RR = 1.2 (1.17, 1.36)	<.00001	Medium
	Effect of 24 h Holter ECG	19 (741/735)	−1	0	0	0	0	RR = 1.2 (1.16, 1.29)	<.00001	Medium
	Adverse reaction	13 (478/474)	−1	0	0	0	0	RR = 0.7 (0.50, 0.98)	.04	Medium
	Atrioventricular block	4 (126/126)	−1	0	0	−1	0	RR = 0.2 (0.04, 0.89)	.04	Low
	Bradycardia	6 (227/220)	−1	0	0	−1	0	RR = 0.5 (0.26, 1.09)	.08	Low
Liu Q (2015)^[23]^	Angina attack frequency	5 (265/265)	−1	−1	0	−1	−1	SMD = −1.41 (−1.97, −0.85)	<.00001	Very low
	Duration of angina	4 (186/186)	−1	0	0	−1	−1	MD = −2.30 (−3.39, −1.21)	<.0001	Very low
	Adverse reaction	6 (347/348)	−1	0	0	−1	−1	RR = 0.84 (0.51, 1.39)	.50	Very low
Li M (2017)^[24]^	Incidence of cardiovascular events	6 (490/419)	−1	0	0	−1	−1	RR = 0.29 (0.19, 0.45)	<.00001	Very low
	ECG improvement	13 (891/749)	−1	0	0	0	−1	RR = 1.23 (1.16, 1.30)	<.00001	Low
Zheng R (2018)^[25]^	Adverse reaction	6 (285/274)	−1	0	0	0	−1	OR = 0..64 (0.39, 1.07)	.17	Low
	Total effective rate	13 (574/555)	−1	0	0	0	−1	RR = 1..22 (1.16, 1.29)	.65	Low
	Cardiac function	2 (82/74)	−1	0	0	−1	−1	RR = 1..22 (1.07, 1.38)	.79	Very low
	Heart rate	2 (97/94)	−1	0	0	−1	−1	MD = −2.25 (−2.61, −1.88)	.46	Very low
	Number of VPB reductions	3 (167/164)	−1	−1	0	−1	−1	MD = −2.03 (−2.41, −1.65)	<.00001	Very low

CI = confidence interval, ECG = electrocardiogram, GRADE = grading of recommendations assessment, development and evaluation, MD = mean difference, RCT = randomized controlled trial, RR = relative risk, SMD = standardized mean difference, TCM = traditional Chinese medicine, VPB = ventricular premature beat, WMD = weighted mean difference.

#### 3.5.1. Outcome index

Eight SRs^[15–18,20,23–25]^ showed a higher total effective rate in the WXKL group than in the control group (GRADE: 1 for medium quality, 6 for low quality, 1 for very low quality). Eight SRs^[15–19,21–23]^ showed that WXKL was superior to the controls in improving the effect of 24-hour Holter electrocardiogram (1 for medium quality, 6 for low quality and 1 for very low quality). Four SRs^[15,19,21,22]^ suggested that WXKL was superior to the control group in improving clinical symptoms, but only one was of medium quality and the rest were of low/very low quality. In addition, there is limited evidence that WXKL is effective in improving electrocardiogram, palpitation, chest tightness and angina.

#### 3.5.2. Adverse events

Adverse events were reported in all 11 SRs, mainly gastrointestinal discomfort in the trial group,^[17,20,22]^ bradycardia, atrioventricular block^[18,19,23,24]^ and occasional sinus arrest^[25]^ in the control group, none of which were serious adverse events. A meta-analysis of 10 SRs for adverse reactions was performed, among which 5 SRs^[15,18,19,23,25]^ showed no significant difference between WXKL and the control group (1 for medium quality and 4 for low quality). The results of the other 5 SRs^[16,17,20–22]^ showed that the incidence of adverse reactions was lower for WXKL than for conventional western drugs (2 for medium quality and 3 for low quality).

### 3.6. Meta-analysis

**Figure 2.**
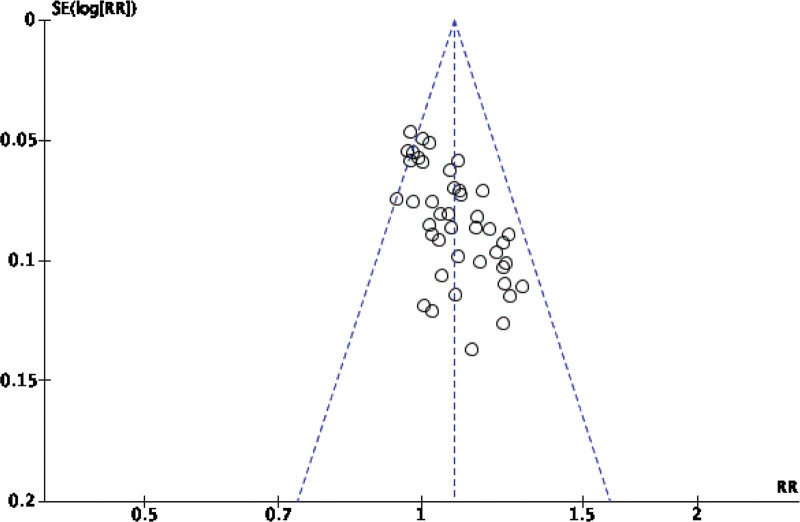
Funnel plot of total effective rate.

**Figure 3.**
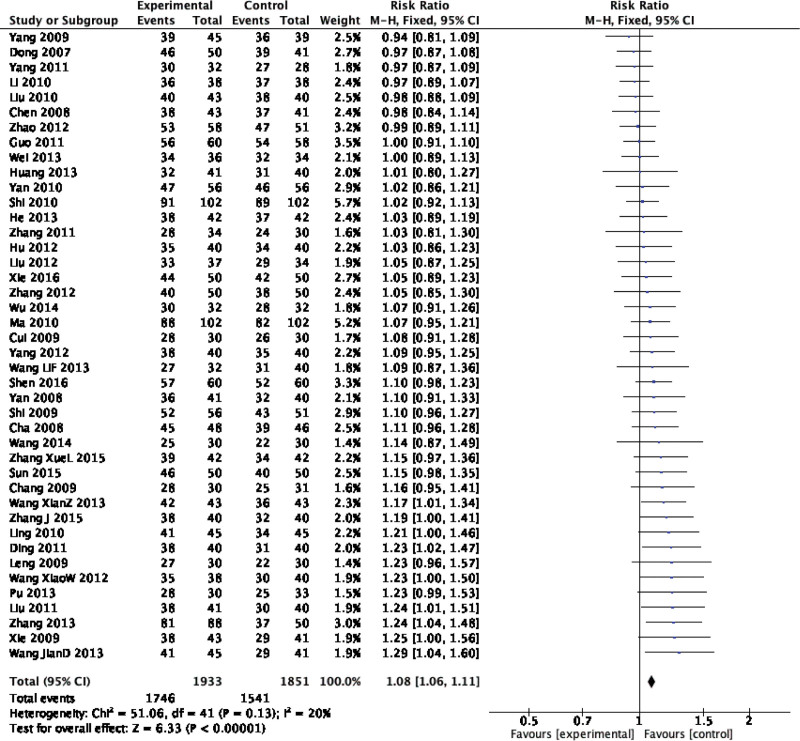
Forest plot of total effective rate.

**Figure 4.**
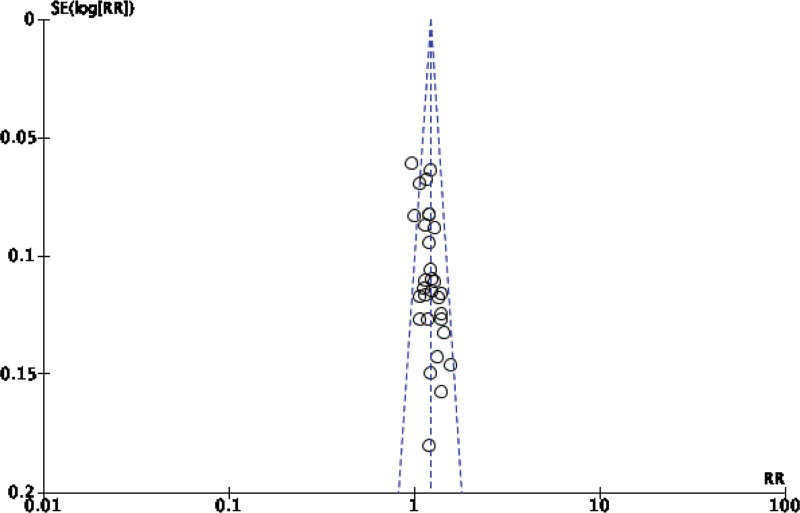
Funnel plot of 24-h Holter ECG efficacy. ECG = electrocardiogram.

**Figure 5.**
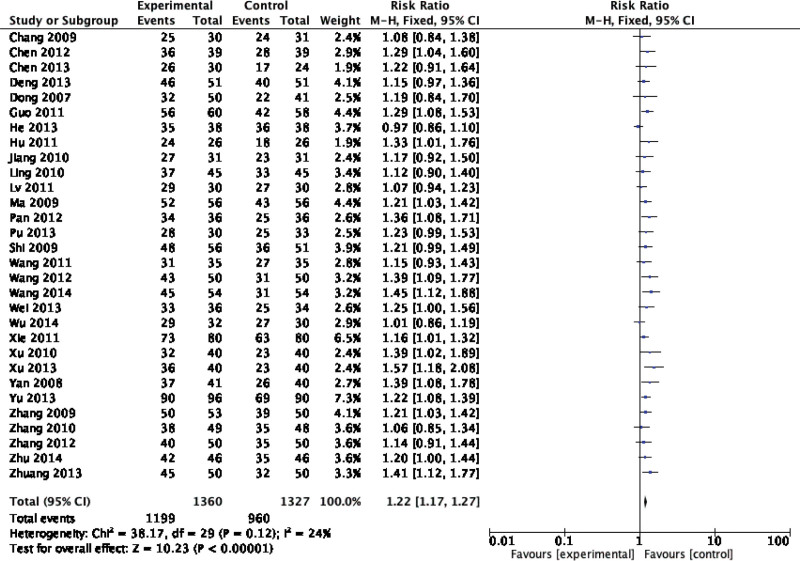
Forest plot of 24-h Holter ECG efficacy. ECG = electrocardiogram.

**Figure 6.**
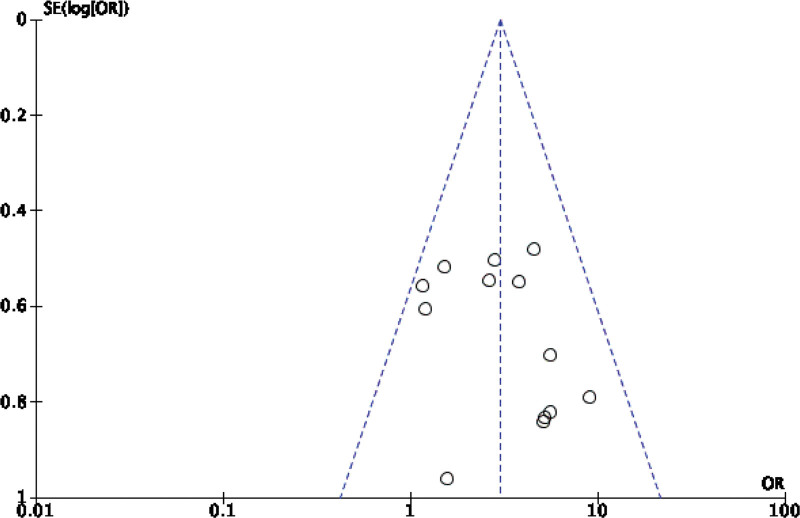
Funnel plot of clinical symptom improvement.

**Figure 7.**
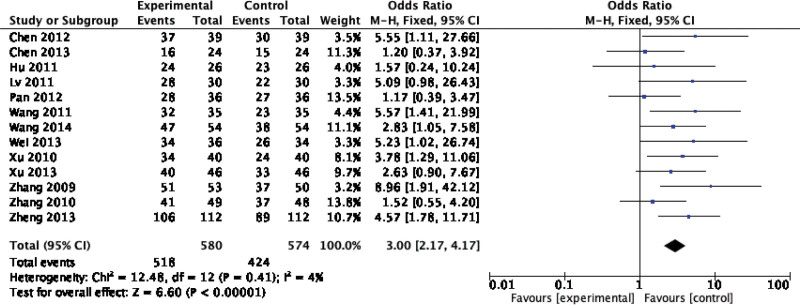
Forest plot of clinical symptom improvement.

**Figure 8.**
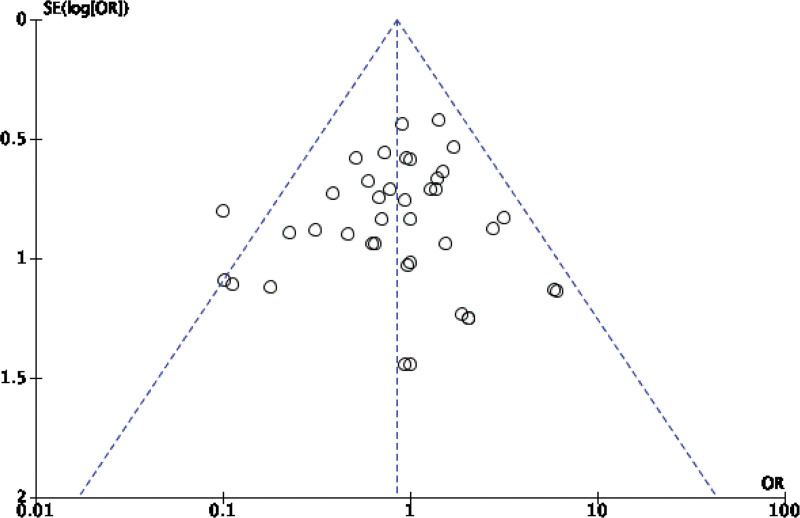
Funnel plot of adverse reaction.

**Figure 9.**
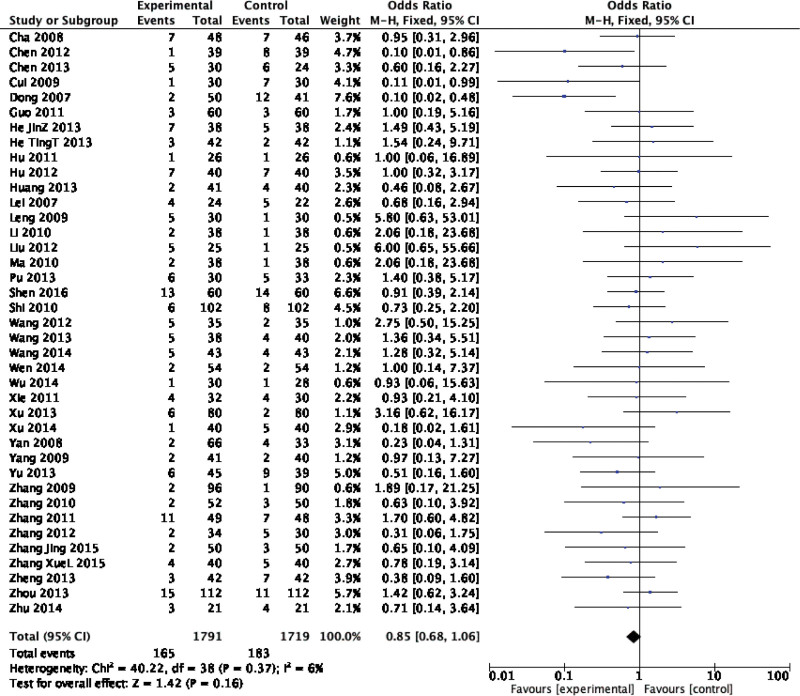
Forest plot of adverse reaction.

Forty-two original RCTs of the 8 SRs evaluated the total effective rate with low heterogeneity (*I*^2^ = 20%, *P =* .13) (see Fig. 2). Using the fixed-effect model combined with effect size, WXKL group was superior to the control group (RR = 1.08, 95% CI: 1.06–1.11, *P* < .00001) (see Fig. 3). Among the 8 SRs, 30 original RCTs reported 24-hour Holter electrocardiogram efficacy with low heterogeneity (*I*^2^ = 24%, *P =* .12) (see Fig. 4) and a fixed-effects model was chosen to combine effect sizes, showing that the WXKL group was superior to the control group (RR = 1.22, 95% CI: 1.17–1.27, *P* < .00001) (see Fig. 5). Of the 4 SRs included in the study, only 13 original RCTs reported clinical symptom improvement, with low heterogeneity (*I*^2^ = 4%, *P =* .41) (see Fig. 6), while fixed-effects models showed that the WXKL group was superior to the control group (RR = 3.00, 95% CI: 2.17–4.27, *P* < .00001) (see Fig. 7). Adverse reactions were reported in 39 original RCTs of 10 SRs and heterogeneity tests showed low heterogeneity (*I*^2^ = 6%, *P* = .37) (see Fig. 8), and the results of the fixed effects model showed no statistical difference between the WXKL group and the control group (RR = 0.85, 95% CI: 0.68–1.06, *P* = .16) (see Fig. 9).

## 4. Discussion

### 4.1. Summary of the main results

This study conducted a data synthesis and descriptive analysis of 11 SRs of WXKL for VPB. The available evidence suggests that WXKL combined with conventional treatment is superior in improving the effect of 24-hour Holter electrocardiogram and clinical symptoms, has good safety, which provides evidence-based information for the clinical application of WXKL for VPB. However, the overall methodological quality of these SRs was low/very low. Individual SR appeared to suggest a benefit of WXKL for VPB; however, the ROBIS assessment showed that the risk of bias was high, which reduced the credibility of the SRs and the GRADE evidence was of insufficient quality (13.04% moderate quality, 52.17% low-quality and 34.78% very low-quality evidence). Therefore, the evidence for the efficacy and safety of WXKL in the treatment of VPB is insufficient for a positive outcome.

### 4.2. Discussion based on results

The results of ROBIS showed that 54.5% of SRs were rated as low risk while 45.5% were rated as high risk. The problems were mainly focused on study identification and selection, data collection and study appraisal and synthesis and findings. The reasons for the low methodological quality of SRs are manifold. Overall, 81.82% of the SRs did not provide a pre-study protocol, which does not determine whether the study plan was strictly followed during SR production, increasing the risk of bias, and does not contribute to the openness and transparency of the study, affecting the rigor and standardization of the SRs.

In total, 91.91% of the SRs did not include a detailed list of exclusions and reasons, which may increase selective bias and affect the overall methodological quality of the systematic evaluation. In addition, none of the SRs explained the reasons for the type of study design included, increasing the risk of bias and reducing the quality of evidence.

According to the results of the quality of evidence grading conducted by GRADE, only 13.04% of the outcomes were rated as moderate and 86.96% of the outcome indicators were rated as low/very low quality of evidence. The reasons for downgrading are mainly as follows: the original studies included in the SRs were unclear in the implementation of randomization, allocation concealment, blinding or did not adequately describe the relevant processes; the original studies did not publish protocols. All of this increased the risk of bias; Factors such as study design, disease diagnostic criteria, disease duration and interventions may contribute to the high heterogeneity of RCTs and reduce the reliability of WXKL for VPB outcomes; The researchers did not provide a reasonable explanation for the high heterogeneity of the results, which increased the inconsistency of SRs; SR retrieval is not comprehensive and lacks searches of gray literature and clinical trial registry platforms. In general, the low quality of evidence is related to the incomplete implementation of study methods by investigators (heterogeneity, precision, publication bias) and is also influenced by the quality of the original studies (flaws in the methodological design of the original RCTs). In future clinical studies, we should focus on the quality of original studies, try to exclude studies with low quality grade and standardize study methods to provide a more scientific and reliable basis for users.

### 4.3. Study on the mechanism WXKL for VPB

WXKL, as a relatively mature proprietary Chinese medicine, has a long history of basic and clinical research. In terms of mechanism of action, numerous studies at home and abroad have shown that WXKL can act on various target genes in human cardiomyocytes and regulate various signaling pathways and ion channels to combat cardiac arrhythmias, as well as inhibit inflammatory responses, reduce myocardial oxidative stress and cardiovascular endothelial damage and improve myocardial ischemia. WXKL has been shown to selectively inhibit late sodium currents,^[26]^ inhibit early and delayed post-depolarization, reduce calcium overload, inhibit ventricular arrhythmias and have a low arrhythmogenic risk and negative inotropic effect. WXKL inhibits the expression of CaMKII^[10]^ and slows the activation of L-type calcium channels.^[27]^ In addition, WXKL significantly improves haemodynamics, inhibits cardiac remodeling and prevents arrhythmias by affecting the MAPK signaling pathway of the inflammation-associated transforming growth factor β-p38/c-Jun amino-terminal kinase and reducing collagen deposition.^[28]^

### 4.4. Suggestions and prospects

Combining all of the assessment results, we made several important recommendations to address the problems: In order to further clarify reliable conclusions about the efficacy and safety of WXKL for VPB, it is necessary for investigators to improve the quality of RCTs and follow the specifications related to clinical trials. According to the uniform standards for clinical trial reporting (CONSORT) and the standards for reporting interventions in pinpoint clinical trials (STRICTA2010), rigorous design, implementation and reporting are performed to reduce the possibility of false positive results or risk of bias; The Authors of SRs should perform comprehensive quality control of SRs based on the A measurement tool to Assess Systematic Reviews 2, ROBIS and GRADE tools to improve the stability and reliability of the evidence; In particular, investigators should consider the ongoing effects of heart stabilizing pellets and focus on the long-term follow-up results of patients.

### 4.5. Strengths and limitation

In recent years, as the systematic evaluation of WXKL for VPB has increased, its quality has been controversial. The current overview is a new approach to collect data on different SRs, reassess the methodological quality and synthesize individual data. To the best of our knowledge, this is the first article to provide a systematic review of SRs about WXKL for VPB. We critically assessed all SRs for methodological quality, risk of bias and quality of evidence and all search and assessment components were guaranteed to be completed by 2 independent reviewers with reliable results. However, there are some limitations of this study, including the limitations of the overview itself and the inadequacy of the SRs. First, there was some publication bias in this study because WXKL is a commonly used Chinese patent medicine, which is more widely used within China, and most of the study results were published in Chinese journals with most of them being positive. Second, the methodological quality of most SRs was not high and the quality of evidence for the results was poor, which could be related to the quality of the original studies. Although the inclusion of lower-quality SRs may introduce some bias, they were nonetheless retained to preserve the objectivity and comprehensive nature of the research. In addition, this study used AMSTAR2 and GRADE to evaluate the quality of methodological and evidence and there were differences in the evaluators’ understanding of the content of the evaluation criteria, which may lead to subjective judgments and bias. This study did not formulate a review plan and no changes were made during the process.

## 5. Conclusion

The efficacy and safety of WXKL for VPB remains to be further demonstrated due to the lack of high-quality evidence in the current SR. The methodological quality of the 11 SRs is low or very low and the conclusions were not highly credible. Therefore, we suggest that clinicians should be careful when making clinical decisions based on these SRs. In the future, well-designed RCTs with large sample sizes will be needed.

## Author contributions

**Conceptualization:** Xia Zhou, Liuying Li.

**Data curation:** Xia Zhou, Wenhua Zhou.

**Formal analysis:** Xia Zhou, Wenhua Zhou, Biao Luo, Daohui Gan.

**Funding acquisition:** Xia Zhou.

**Methodology:** Biao Luo, Daohui Gan, Xiaohan Wu, Fengya Zhu.

**Project administration:** Fengya Zhu.

**Resources:** Lvyu Deng, Fengya Zhu.

**Software:** Lvyu Deng, Xiaohan Wu.

**Visualization:** Liuying Li.

**Writing – original draft:** Xia Zhou.

**Writing – review & editing:** Fengya Zhu, Liuying Li.

## Supplementary Material


