# Supplementary material for: Efficacy and safety of Wenxin Keli for ventricular premature beat: An overview of systematic reviews and meta-analyses
Source: Medicine (Baltimore). 2026 Jan 16;105(3):e47265. doi: 10.1097/MD.0000000000047265 (PMC12826144; doi:10.1097/MD.0000000000047265)
Supplement: Supplementary file 1 [file medi-105-e47265-s001.pdf]

Excluded list

Citation	Reason for exclusion
Chen Y, Nie S, Gao H, Sun T, Liu X, Teng F, Xing Y, Chen W, Zhang Z, Gao Y, Wang J, Xing Y, Shang H. The effects of wenxin keli on p-wave dispersion and maintenance of sinus rhythm in patients with paroxysmal atrial fibrillation: a meta-analysis of randomized controlled trials. Evid Based Complement Alternat Med. 2013;2013:245958.	The disease did not meet the inclusion criteria
Meng LL, Huang W. A meta-analysis of wenxin granule and metoprolol for the treatment of coronary heart disease and arrhythmia. Medicine (Baltimore). 2022 Sep 2;101(35):e30250.	The disease did not meet the inclusion criteria
Shi S, Chu Y, Jia Q, Hu Y. Comparative efficacy and safety of wenxin granule combined with antiarrhythmic drugs for atrial fibrillation: A protocol for a systematic review and network meta-analysis. Medicine (Baltimore). 2021 Jan 22;100(3):e24434.	The disease did not meet the inclusion criteria
Wang Y, Liu Y, Yan X, Wang D. An Overview of Systematic Reviews on the Effectiveness of Wenxin Keli in the Treatment of Atrial Fibrillation. Evid Based Complement Alternat Med. 2022 May 30;2022:6973151.	The disease did not meet the inclusion criteria
Yuan Y, Xiong XJ, Li DD, Li HX, Fu JP, Wu HL. Efficacy and Safety of Wenxin Granules and Propafenone in Treatment of Atrial Premature Beats: A Systematic Review and Meta-Analysis. Evid Based Complement Alternat Med. 2020 Jul 23;2020:3961091.	The disease did not meet the inclusion criteria
Wang X, Wang Y, Feng X, Lu Y, Zhang Y, Wang W, Zhu W. Systematic review and meta-analysis of randomized controlled trials on Wenxin keli. Drug Des Devel Ther. 2016 Nov 14;10:3725-3736.	The disease did not meet the inclusion criteria
Liu CY, Lu J, Li JT, Lu JG. Meta-analysis of ventricular arrhythmias after myocardial infarction treated with heart-stabilizing granules[J]. Hunan Journal of Traditional Chinese Medicine,2019,35(01):104-109.	The disease did not meet the inclusion criteria
ZHANG MM, CHEN ZQ, XING ZY, REN HJ, WANG YX. Meta-analysis of heart-stabilizing granules in the treatment of tachyarrhythmias[J]. Chinese Medicine Research,2021,34(03):43-48.	The disease did not meet the inclusion criteria
Wei RL, Wang ZF, Ma XC, Zhang Q, Cui X, Xie YM. Comprehensive clinical evaluation of heart stabilizing granules in the treatment of cardiac arrhythmias (Qi and Yin deficiency evidence)[J]. Chinese Journal of Traditional Chinese Medicine,2021,46(23):6068-6077.	The disease did not meet the inclusion criteria
Wang ZB, Zhao YJ, Zhou FC, Zhu YH, Liu L, Feng C, Chen ZL. Meta-analysis of clinical efficacy of heart-stabilizing granules compared with western antiarrhythmic drugs in the treatment of atrial fibrillation[J]. Chinese Journal of Traditional Chinese Medicine,2019,44(11):2379-2389.	The disease did not meet the inclusion criteria
Xin Y, Dai YY, Yang HX, Zhang XW, Li B. Systematic evaluation of efficacy and safety of heart stabilizing granules in the treatment of chronic heart failure combined with atrial fibrillation[J]. Chinese Journal of Traditional Chinese Medicine,2019,44(23):5198-5206.	The disease did not meet the inclusion criteria

Li YH, Zhu HJ. Systematic evaluation of a randomized controlled trial of Ginseng Yangxin Capsules for the treatment of premature ventricular contractions[J]. Chinese Clinical Pharmacology and Therapeutics,2009,14(08):915-923.	Interventions did not meet inclusion criteria
Zhang P, Wu LQ, Yang HJ, Li B, Xu FQ, Ma XC. Systematic evaluation of a randomized controlled trial of Ginseng Yangxin Capsules for the treatment of premature ventricular contractions in coronary artery disease[J]. Journal of Integrative Medicine and Cardiovascular Diseases,2015,13(04):460-463.	Interventions did not meet inclusion criteria
Shi S-N, Deng Y-P, Liu J-H, Wang P-L. Meta-analysis of Chinese patent medicine combined with beta-blockers for ventricular premature beats in coronary artery disease[J]. Chinese Journal of Traditional Chinese Medicine Information,2022,29(05):30-37.	Interventions did not meet inclusion criteria
Wu HF, Guo ZQ, Li XX, Kuang W, Zhang JQ, Xu QW. Meta-analysis of the effectiveness and safety of combined Chinese and Western medicine in the treatment of ventricular precontraction[J]. Journal of Integrative Medicine and Cardiovascular Diseases,2021,19(08):1267-1274.	Interventions did not meet inclusion criteria
WANG JR,ZHU MJ,WANG XL,YU Rui,PENG GGo,WANG YG. Systematic evaluation of the randomized controlled trial of Tongguoyangxinwan for the treatment of premature ventricular contractions[J]. Chinese Pharmacology and Clinical Practice,2020,36(02):215-220.	Interventions did not meet inclusion criteria
Wang F, Xu H Y, Du W T, Zhang Y Y, Zhang N, Liu P. Systematic evaluation of different prescriptions to benefit qi and nourish yin in the treatment of tachyarrhythmic premature ventricular contractions with qi and yin deficiency evidence[J]. Chinese Traditional Chinese Medicine Acute Care,2016,25(01):54-57+75.	Interventions did not meet inclusion criteria
Xu JG, Cheng XY, Ge L. Meta-analysis on the efficacy and safety of Ginseng Yangxin Capsules in the treatment of chronic heart failure combined with premature ventricular contractions[J]. Anhui Medicine,2018,22(01):122-126.	Interventions did not meet inclusion criteria
Zhang Y, Yuan ZD. Meta-analysis of the effectiveness and safety of ginseng yangxin capsule combined with betalactone in the treatment of premature ventricular contractions[J]. Journal of Doubtful Diseases,2016,15(06):629-633.	Interventions did not meet inclusion criteria
Leong H, Jiang C G, Jiang X T, Wen J M. Meta-analysis and trial sequential analysis of the efficacy of heart stabilizing granules combined with metoprolol in the treatment of hypertensive heart disease combined with premature ventricular contractions[J]. Journal of Integrative Medicine and Cardiovascular Diseases,2019,17(02):161-169.	Contains two diseases
Liao G P, Deng F W, Sun D G, Hu J H, Lin J M, Yin X X. Meta-analysis on the efficacy and safety of heart-stabilizing granules in the treatment of viral myocarditis combined with premature ventricular contractions[J]. Strait Pharmacology,2019,31(08):163-166.	Contains two diseases
Liang YY, Yao XB. Meta-analysis of heart stabilizing granules combined with amiodarone in the treatment of heart failure combined with arrhythmias[J]. Journal of Integrative Medicine and Cardiovascular Diseases,2017,15(16):2006-2009.	Contains two diseases

Li YQ. Meta-analysis of the effectiveness and safety of amiodarone combined with heart stabilizing granules in the treatment of heart failure combined with arrhythmias[J]. Journal of Clinical Rational Drug Use,2022,15(10):1-4.	Contains two diseases
Shi S, Geng YT, HY, Song QQ, Du B, Feng R. Systematic evaluation of heart stabilizing granules combined with bisoprolol in the treatment of heart failure combined with arrhythmias[J]. Chinese Journal of Experimental Formulary,2016,22(15):216-219.	Contains two diseases
Wei L, Cao H, Huang C X, Tang Y H. Systematic evaluation of heart stabilizing granules combined with metoprolol in the treatment of ventricular arrhythmias combined with coronary artery disease[J]. Hainan Medicine,2014,25(05):752-757.	Contains two diseases
Wang YH. Systematic evaluation of heart stabilizing granules combined with metoprolol in the treatment of ventricular arrhythmias combined with coronary artery disease[J]. Northern Pharmacy,2016,13(05):14-16.	Contains two diseases
Hong Najiao. Systematic evaluation of heart stabilizing granules combined with metoprolol in the treatment of ventricular arrhythmias combined with coronary artery disease[J]. Knowledge of Cardiovascular Disease Prevention and Control (Academic Edition),2015(22):68-69.	Contains two diseases
Lin Kaili, Yan Kui Po, Sun Yan Qin et al. Meta-analysis of heart stabilizing granules in the treatment of cardiac arrhythmias[C]//Professional Committee of Evidence-Based Medicine, Chinese Society of Integrative Medicine. Conference Materials of the 9th Symposium on Evidence-Based Approaches to Traditional Chinese Medicine/Western Medicine. 2015:190-193.	Conference article
Zhang P, Li B, Xu FQ et al. Systematic evaluation of a randomized controlled trial of Ginseng Yangxin Capsules compared with Heart Rhythm Tablets for the treatment of ventricular premature beats in coronary heart disease[C]//Chinese Physicians Association, Chinese and Western Medicine Physicians Branch, Fujian University of Traditional Chinese Medicine. 2012.	Conference article
Sun LHn, Zhang C, Yan H et al. Meta-analysis of the efficacy and safety of heart-stabilizing granules in the treatment of premature ventricular contractions[C]//Chinese Physicians Association,Chinese and Western Medicine Physicians Association.2014:1.	Conference article
Xiang Jinsong, Liu YJ, Fan Huai C et al. Systematic evaluation of a randomized controlled trial of heart-stabilizing granules for the treatment of premature ventricular contractions[J]. Liaoning Journal of Traditional Chinese Medicine,2009,36(03):380-381.	Descriptive analysis
Wang S Y, Lu Y, Feng X Y, Wang Y, Wang Xuan, Zhu W T. Pharmacoeconomic evaluation of heart stabilizing granules for cardiovascular diseases based on Meta-analysis[J]. China Pharmacy,2017,28(05):591-595.	Descriptive analysis

PubMed database

#1	((((((((Ventricular premature beat[MeSH Terms]) OR (Premature ventricular contraction[MeSH Terms])) OR (VPB[Text Word])) OR (PVC[Text Word])) OR (Ventricular premature beat syndrome[Text Word])) OR (Ventricular Premature Complexes[Text Word])) OR (Ventricular premature contraction[Text Word])))
#2	(((((Wenxin Keli) OR (Wenxin-Keli)) OR (Wenxin)) OR (WXKL)) OR (Wenxin particle)) [[All Fields]
#3	((systematic review[Text Word]) OR (systematic evaluation[Text Word])) OR (meta analysis[Text Word])
#4	#1 AND #2 AND #3

Embase database

#1	'Ventricular premature beat':ti,ab,kw OR 'Premature ventricular contraction':ti,ab,kw OR 'Ventricular premature beat syndrome':ti,ab,kw OR chd:ti,ab,kw OR 'Ventricular Premature Complexes':ti,ab,kw OR 'Ventricular premature contraction':ti,ab,kw
#2	'Wenxin Keli':ti,ab,kw OR 'Wenxin-Keli':ti,ab,kw OR 'Wenxin':ti,ab,kw
#3	'systematic review':ti,ab,kw OR 'systematic evaluation':ti,ab,kw OR 'meta analysis':ti,ab,kw
#4	#1 and #2 and #3

Cochrane library database

#1	(Wenxin Keli):ti,ab,kw
#2	(Wenxin-Keli):ti,ab,kw
#3	(Wenxin):ti,ab,kw
#4	(WXKL):ti,ab,kw
#5	(Wenxin particle):ti,ab,kw
#6	(wenxinkeli):ti,ab,kw
#7	#1 OR #2 OR #3 OR #4 OR #5 OR #6
#8	Ventricular premature beat: MeSH
#9	Premature ventricular contraction:MeSH
#10	(Ventricular premature beat syndrome):ti,ab,kw
#11	(VPB):ti,ab,kw

#12	(PVC):ti,ab,kw
#13	(Ventricular Premature Complexes):ti,ab,kw
#14	(Ventricular premature contraction):ti,ab,kw
#15	#8 OR #9 OR #10 OR #11 OR #12 OR #13 OR #14
#16	(systematic review):ti,ab,kw
#17	(systematic evaluation):ti,ab,kw
#18	(meta-analysis):ti,ab,kw
#19	#16 OR #17 OR #18
#20	#7 AND #15 AND #19
